# Murine cardiac fibrosis localization using adaptive Bayesian cardiac strain imaging in vivo

**DOI:** 10.1038/s41598-022-12579-6

**Published:** 2022-05-20

**Authors:** Rashid Al Mukaddim, Ashley M. Weichmann, Rachel Taylor, Timothy A. Hacker, Thomas Pier, Joseph Hardin, Melissa Graham, Carol C. Mitchell, Tomy Varghese

**Affiliations:** 1 Medical Physics, University of Wisconsin (UW)-Madison, Madison, USA; 2 Small Animal Imaging and Radiotherapy Facility, UW-Madison, Madison, USA; 3 Cardiovascular Physiology Core Facility, UW-Madison, Madison, USA; 4 Experimental Animal Pathology Lab, UW-Madison, Madison, USA; 5 Comparative Pathology Laboratory, Research Animal Resources and Compliance (RARC), UW-Madison, Madison, USA; 6 Medicine/Division of Cardiovascular Medicine, UW-Madison, Madison, USA

**Keywords:** Medical research, Engineering

## Abstract

An adaptive Bayesian regularized cardiac strain imaging (ABR-CSI) algorithm for in vivo murine myocardial function assessment is presented. We report on 31 BALB/CJ mice (n = 17 females, n = 14 males), randomly stratified into three surgical groups: myocardial infarction (MI, n = 10), ischemia–reperfusion (IR, n = 13) and control (sham, n = 8) imaged pre-surgery (baseline- BL), and 1, 2, 7 and 14 days post-surgery using a high frequency ultrasound imaging system (Vevo 2100). End-systole (ES) radial and longitudinal strain images were used to generate cardiac fibrosis maps using binary thresholding. Percentage fibrotic myocardium (PFM) computed from regional fibrosis maps demonstrated statistically significant differences post-surgery in scar regions. For example, the MI group had significantly higher PFM_Radial_ (%) values in the anterior mid region (*p* = 0.006) at Day 14 (n = 8, 42.30 ± 14.57) compared to BL (n = 12, 1.32 ± 0.85). A random forest classifier automatically detected fibrotic regions from ground truth Masson’s trichrome stained histopathology whole slide images. Both PFM_Radial_ (*r* = 0.70) and PFM_Longitudinal_ (*r* = 0.60) results demonstrated strong, positive correlation with PFM_Histopathology_ (*p* < 0.001).

## Introduction

Coronary heart disease (CHD) including myocardial infarction (MI) due to ischemia is the number one cause of mortality worldwide according to the American Heart Association annual statistical update 2021^1^ and it is estimated that CHD accounts for 13% of all deaths annually in 2018^1^. One of the changes that can be seen with myocardial ischemia is myocardial remodeling. Myocardial remodeling occurs when the myocardium undergoes a series of morphological changes after an ischemic event (e.g., change in mass and geometry, scar formation)^2^. Animal models of MI and ischemia–reperfusion (IR) have been instrumental in gaining insights on cardiac remodeling^3,4^. In particular, murine models of CHD have been routinely utilized in pre-clinical research due to their lower cost, convenience and ease of genetic alteration^5^. To understand the anatomical and physiological changes associated with these models, accurate and reproducible non-invasive techniques for measuring cardiac physiology are of utmost importance^6^. One key aspect of non-invasive monitoring is cardiac fibrosis localization as it is related to myocardial viability and loss of functional myocardial tissue post MI and IR^6,7^. Furthermore, focal fibrosis causes enhanced stiffness which in turn reduces segmental myocardial velocity^6^. Non-invasive cardiac imaging such as ultrasound based cardiac strain imaging (CSI)^8–10^ can play a pivotal role in studying murine cardiac fibrosis in vivo^4,5^.

Cardiac strain imaging utilizing the ultrasound envelope or radio-frequency (RF) echocardiography data, has shown widespread applicability in both clinical and pre-clinical studies as it can be performed during a conventional echocardiographic examination^8,9,11–15^. Envelope-based CSI (known as speckle tracking echocardiography [STE])^16–18^ has been used extensively in humans^19^ and is available on most clinical US systems^20^ and also been utilized to study murine cardiac fibrosis in vivo^7^. For example, Peng et al. reported associations between STE-derived radial strain and histopathology derived cardiac fibrosis measures^7^. Bhan et al*.* showed that STE-derived segmental radial and longitudinal strain were correlated with MRI-derived infarct size^21^. However, CSI using ultrasound RF data is more sensitive to subtle myocardial motion abnormalities compared to envelope-based STE^16–18^. RF-based CSI^8,9^ has been shown to be feasible in humans^22,23^ and large animal models^24,25^. However, very few RF-based CSI studies have been performed in mice due to this method’s increased sensitivity to signal decorrelation associated with large deformations^26,27^. This finding is thought to occur in murine models due their small size and rapid heart rate^21^. Higher heart rates result in increased RF signal decorrelation and additional out-of-plane scatterer motion due to imaging the complex 3-D cardiac deformation and echo twist in 2-D thus degrading the quality of CSI.

To address the challenges associated with RF-based CSI in murine models, we have recently developed an adaptive Bayesian regularized cardiac strain imaging (ABR-CSI) algorithm. Regularization provides safeguards against motion estimation inaccuracies associated with large deformations and increased RF signal decorrelation. ABR-CSI dynamically controls regularization using local RF data statistics and has demonstrated improved performance over conventional block matching algorithms^28^ in cardiac finite-element analysis (FEA) simulation studies^29^. Additionally, feasibility of ABR-CSI for in vivo experimental studies has been shown on a limited number (n = 10) of healthy murine hearts^29–32^. However, ABR-CSI for murine models of myocardial injury in vivo in a larger cohort has not been investigated. We hypothesize that ABR-CSI can be used to localize cardiac fibrosis in murine injury models of myocardial ischemia and ischemia–reperfusion in vivo*.* Therefore, the primary objective of this paper is to provide evidence demonstrating reliability of cardiac fibrosis measurements using ABR-CSI in murine heart in vivo and evaluate its utility in experimental studies.

This paper reports on two main contributions. First, the demonstration of serial in vivo evaluation of murine cardiac fibrosis derived from radial and longitudinal strain images estimated with ABR-CSI. Second, we validate in vivo findings against ground truth fibrosis measures extracted from ex vivo Masson’s trichrome (MT) stained histopathological whole slide images (WSI).

## Results

### An adaptive Bayesian regularized cardiac strain imaging algorithm for in vivo cardiac fibrosis localization

**Figure 1:**
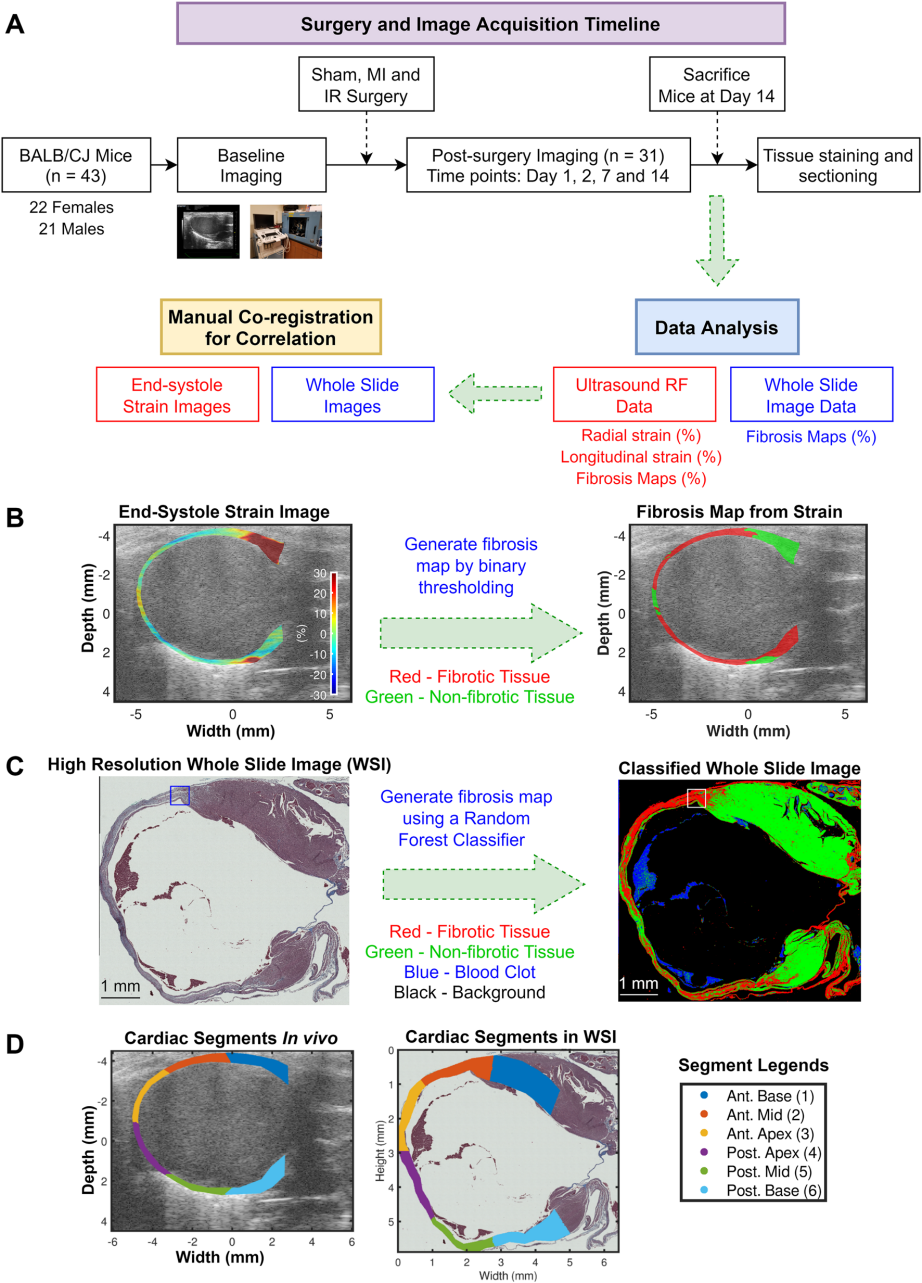
Cardiac fibrosis localization using adaptive Bayesian regularized cardiac strain imaging (ABR-CSI) in vivo. (**A**) Experimental protocol for histopathological validation of proposed ABR-CSI in murine models of myocardial infarction (MI) and ischemia–reperfusion (IR). High frequency ultrasound RF data collected pre- and 1, 2, 7 and 14 days post-surgery were used for strain estimation. (**B**) Method for estimating cardiac fibrosis using cumulative ES strain images in vivo. (**C**) Method for estimating cardiac fibrosis using high resolution WSI ex vivo. A random forest classifier detects four classes in the raw WSI: fibrotic, non-fibrotic regions, blood clots and background. (**D**) Cardiac segment definitions for regional analysis. *Ant.* anterior and *Post.* posterior.

A longitudinal study with murine injury models of myocardial ischemia and ischemia reperfusion was designed to compare in vivo strain variations against ground truth histopathology WSI (Fig. 1A). Cardiac fibrosis maps were generated by applying binary thresholding on end-systole (ES) radial and longitudinal strain images (Fig. 1B). Cardiac fibrosis maps classify the entire myocardium into fibrotic (red) and non-fibrotic (green) regions. A random forest classifier trained using QuPath^33^ generated the fibrosis maps from the ex vivo high resolution WSI data (Fig. 1C). Finally, following American Heart Association (AHA) recommendations^34^, cardiac segmentation was performed by dividing the myocardium into six equal segments in corresponding in vivo ultrasound and ex vivo WSI data for regional correlation analysis (Fig. 1D).

### Temporal progression of ES strain images allows monitoring of cardiac remodeling in murine models of MI, IR and Sham in vivo

**Figure 2:**
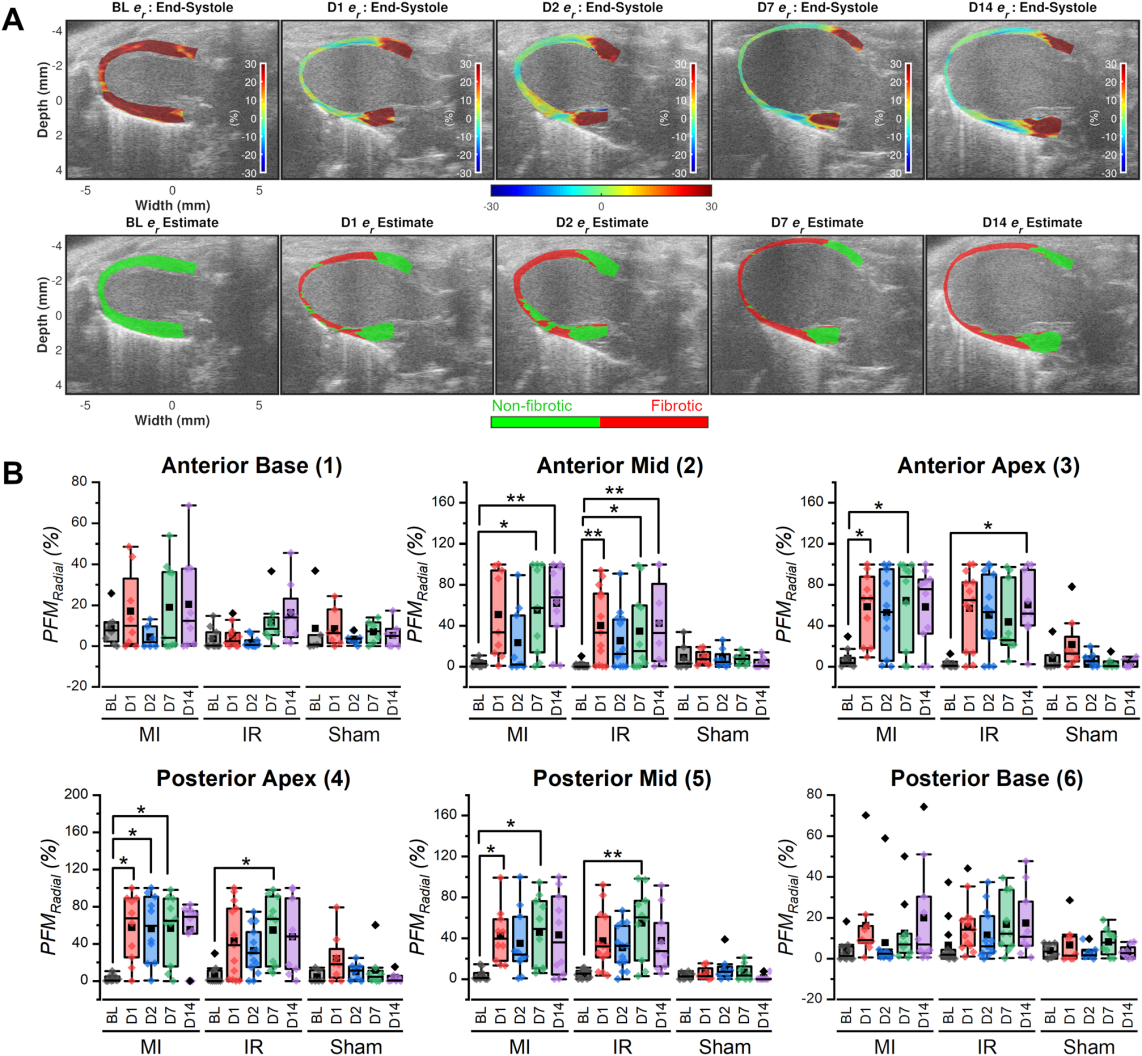
Monitoring cardiac remodeling using ES radial strain images. (**A**) *Top row:* Temporal progression of ES radial strain images from BL to Day 14 (D14) in a female BALB/CJ mouse after MI surgery. Strain display dynamic range is from − 30 to + 30%. Positive strain value (red) = myocardial wall thickening, negative strain value (blue) = myocardial wall thinning and zero strain value (light green) = no wall motion. *Bottom row:* Cardiac fibrosis maps estimated using ES radial strain images presented on the top row. Red = fibrotic region, green = non-fibrotic region. (**B**) *Top row:* Box and whisker plots demonstrating temporal variation and inter-group comparison [MI (n = 10), IR (n = 13) and Sham (n = 8)] of percentage fibrotic myocardium (PFM) estimated from radial strain (*PFM*_*Radial*_) in the anterior myocardial segments. *Bottom row:* Box and whisker plots demonstrating temporal variation and inter-group comparison (MI, IR and Sham) of *PFM*_*Radial*_ in the posterior myocardial segments. Friedman’s test with Bonferroni–Dunn post hoc test was used. Symbol definitions: BL—Baseline, D1—Day 1, D2—Day 2, D7—Day 7, D14—Day 14, Squares—mean, Diamonds—outliers, whiskers—1.5 × IQR (Inter-quartile range), box—25th to 75th percentiles, *p < 0.05, **p < 0.01, ***p < 0.001.

Figure 2 summarizes our results for monitoring post-surgery cardiac remodeling using ES radial strain images. At baseline (BL), the mouse presented with normal LV function having a uniformly red strain map at ES (myocardial wall thickening) and a uniformly green fibrosis map indicating absence of fibrotic regions (Fig. 2A). Post-surgery ES radial strain images showed a marked reduction or sign reversal of strain magnitudes (light green or blue regions) in the apical segments when compared to BL. The corresponding fibrosis maps enable concise qualitative visualization and localization of the area of fibrosis indicated using red color (Fig. 2A, bottom row). Note that the sham group preserved normal LV function for all segments with low PFM_Radial_ (%) values at all image acquisition time points. For example, the average anterior apical PFM_Radial_ (%) values for the sham group at BL (n = 7) and Day 14 (n = 7) were 7.91 ± 5.55 and 4.09 ± 1.47 with no statistically significant difference (non-significant Freidman’s test result; Chi-square = 5.05, p = 0.4, Supplementary Table S3). Both MI and IR showed low PFM_Radial_ (%) at BL with substantial increases post-surgery. Qualitative observation of Fig. 2B shows that apical and mid segments had a relatively larger increase in PFM_Radial_ (%) values compared to basal segments. For example, the MI group had higher PFM_Radial_ (%) values in the anterior apical segment at Day 14 (n = 10, 58.29 ± 11.94) compared to BL (n = 9, 7.83 ± 3.23). Even though Freidman’s test was significant for the anterior apical segment in the MI group (Chi-square = 15.58, p = 0.013), Bonferroni–Dunn post hoc test did not show a statistically significant difference between BL and D14 results (p = 0.736, Supplementary Table S2). On the other hand, the mean anterior basal PFM_Radial_ (%) values showed relatively lower increase from BL (n = 8, 8.63 ± 2.90) to Day 14 (n = 9, 20.43 ± 7.79) with non-significant Freidman’s test result (Chi-square = 7.56, p = 0.109, Supplementary Table S2).

**Figure 3:**
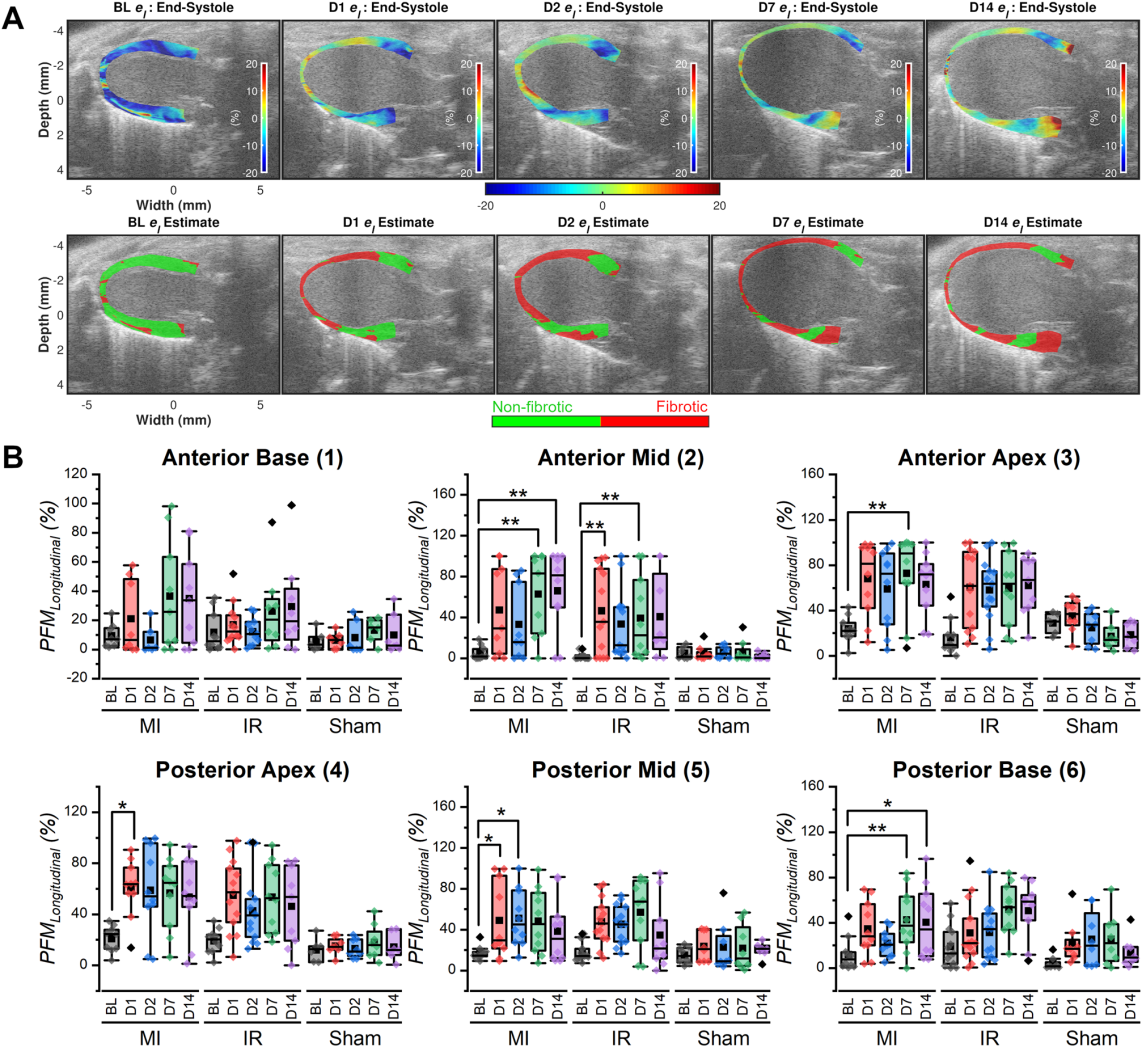
Monitoring cardiac remodeling using ES longitudinal strain images. (**A**) *Top row:* Temporal progression of ES longitudinal strain images from BL to Day 14 (D14) in a female BALB/CJ mouse after MI surgery. Strain display dynamic range is from − 20 to + 20%. Negative strain value (blue) = myocardial wall shortening, positive strain value (red) = myocardial wall elongation and zero strain value (light green) = no wall motion. *Bottom row:* Cardiac fibrosis maps estimated using the ES longitudinal strain images presented on the top row. Red = fibrotic region, green = non-fibrotic region. (**B**) *Top row:* Box and whisker plots demonstrating temporal variation and inter-group comparison [MI (n = 10), IR (n = 13) and Sham (n = 8)] of PFM estimated from longitudinal strain (*PFM*_*Longitudinal*_) in the anterior myocardial segments. *Bottom row:* Box and whisker plots demonstrating temporal variation and inter-group comparison (MI, IR and Sham) of *PFM*_*Longitudinal*_ in the posterior myocardial segments. Friedman’s test with Bonferroni–Dunn post hoc test was used. Symbol definitions: BL—Baseline, D1—Day 1, D2—Day 2, D7—Day 7, D14—Day 14, Squares—mean, Diamonds—outliers, whiskers—1.5 × IQR (Inter-quartile range), box—25th to 75th percentiles, *p < 0.05, **p < 0.01, ***p < 0.001.

Results quantifying cardiac fibrosis using ES longitudinal strain images are presented in Fig. 3. This mouse had normal LV function along the longitudinal direction at BL with a predominantly blue ES strain image indicating myocardial wall shortening (Fig. 3A). In post-surgery strain images, there was a marked reduction or sign reversal of strain magnitudes (light green or red) in the myocardium. Regions with altered strain progressively increased from apical segments at Day 1 to mid and basal segments by Day 14. The sham group presented with lower segmental PFM_Longitudinal_ at all post-surgery time points compared to the MI and IR groups that was confirmed with the Kruskal–Wallis test (Supplementary Tables S13–S18). For example, for the anterior apical segment at D14, PFM_Longitudinal_ (%) values for sham, MI and IR groups were 18.56 ± 4.16 (n = 7), 63.09 ± 8.76 (n = 10) and 62.10 ± 8.70 (n = 9) with statistically significant difference with the sham group (Bonferroni–Dunn post hoc test; Supplementary Table S15) compared to MI and IR (Sham vs IR, *p* = 0.014; Sham vs MI, *p* = 0.009, Supplementary Table S6). Furthermore, none of the segments showed statistically significant differences in PFM_Longitudinal_ (%) values during the experimental period (e.g., segment 1: Freidman’s test Chi-square = 0.48, p = 0.975). Both MI and IR showed low PFM_Longitudinal_ (%) values at BL with considerable increase post-surgery. For the MI group, the mean anterior apical PFM_Longitudinal_ (%) values increased from BL (n = 9, 23.77 ± 3.94) to Day 14 (n = 10, 63.09 ± 8.76) however without statistical significance (p = 0.526, Supplementary Table S5). Similarly, for the IR group, the anterior apical PFM_Longitudinal_ (%) values differed between BL (n = 10, 16.17 ± 4.99) and Day 14 (n = 9, 62.10 ± 8.70) without statistical significance (p = 1.00, Supplementary Table S4).

### Cardiac strain imaging identifies regions of fibrosis and correlates with MT stained digital histopathology WSI data

**Figure 4:**
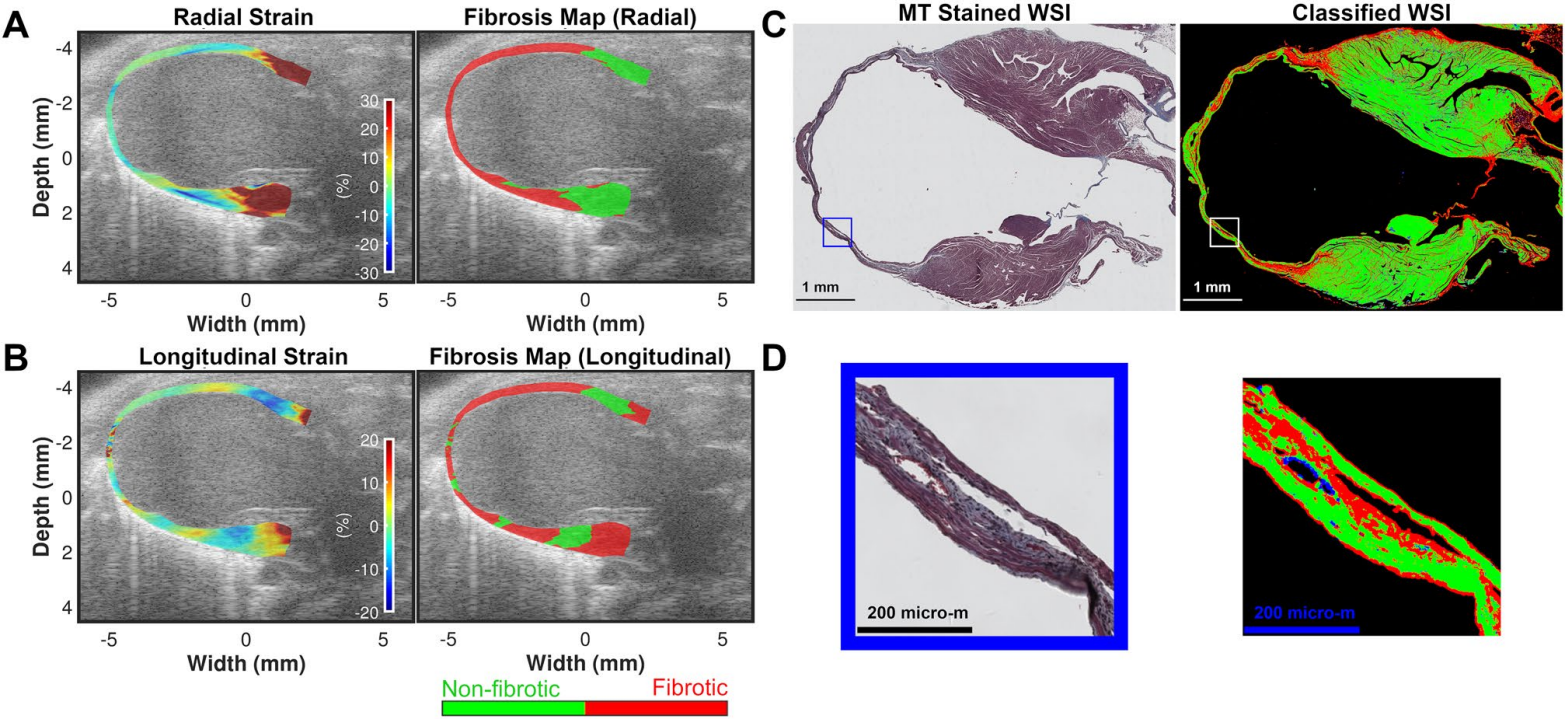
Qualitative comparison between cardiac fibrosis maps estimated using strain images in vivo and WSI data ex vivo in a female BALB/CJ mouse after myocardial infarction surgery. (**A**) Left panel: Cumulative ES radial strain image at Day 14. Right panel: Corresponding cardiac fibrosis map. (**B**) Cumulative ES longitudinal strain image at Day 14. Right panel: Corresponding cardiac fibrosis map. Red = fibrotic region, green = non-fibrotic region. (**C**) Left panel: MT stained WSI. Right panel: Corresponding classified image generated using a random forest classifier. Red = fibrotic region, green = non-fibrotic region, blue = blood clot, black = background. (**D**) Left panel: A magnified region-of-interest extracted from the WSI image demonstrating collagen deposition in the fibrotic regions. Right panel: Corresponding classified image using the random forest classifier.

Figure 4 shows qualitative comparisons between cardiac fibrosis maps estimated using strain images in vivo and WSI data ex vivo in a female BALB/CJ mouse after myocardial infarction surgery. Radial strain identified apical segments primarily as fibrotic with extension into the mid segment while basal segments were identified as non-fibrotic (Fig. 4A). Conversely, longitudinal strain indicates nearly the entire myocardium to be fibrotic except for small regions in the anterior and posterior basal segments (Fig. 4B) with relatively higher estimation of fibrotic area in the posterior basal segments compared to the radial strain results. This qualitative observation corroborates our results presented in Figs. 2 and 3 and Supplementary Tables S2 and S5 respectively. For the MI group, PFM_Radial_ (%) values at the posterior basal segments did not differ significantly between BL and D14 (p = 0.237, Supplementary Table S2) while corresponding PFM_Longitudinal_ (%) values showed a significant difference (p = 0.011, Supplementary Table S5). Thinning of the apical wall (Fig. 4C) with the presence of collagen fibers (observe blue staining in the magnified region presented in Fig. 4D) were seen in the MT stained histopathology WSI. The corresponding classified image confirms that the region of fibrosis is primarily located in the apical segments with an extension into the mid segments (Fig. 4C,D). These results demonstrate that the radial strain fibrosis map has better qualitative agreement with the ground truth fibrosis map compared to the longitudinal strain fibrosis map. A similar result was observed for a mouse with smaller MI (Supplementary Fig. S1) where the radial strain fibrosis map reliably located the MI location resulting in better qualitative agreement with the histopathology fibrosis map compared to the longitudinal strain fibrosis map.

**Figure 5:**
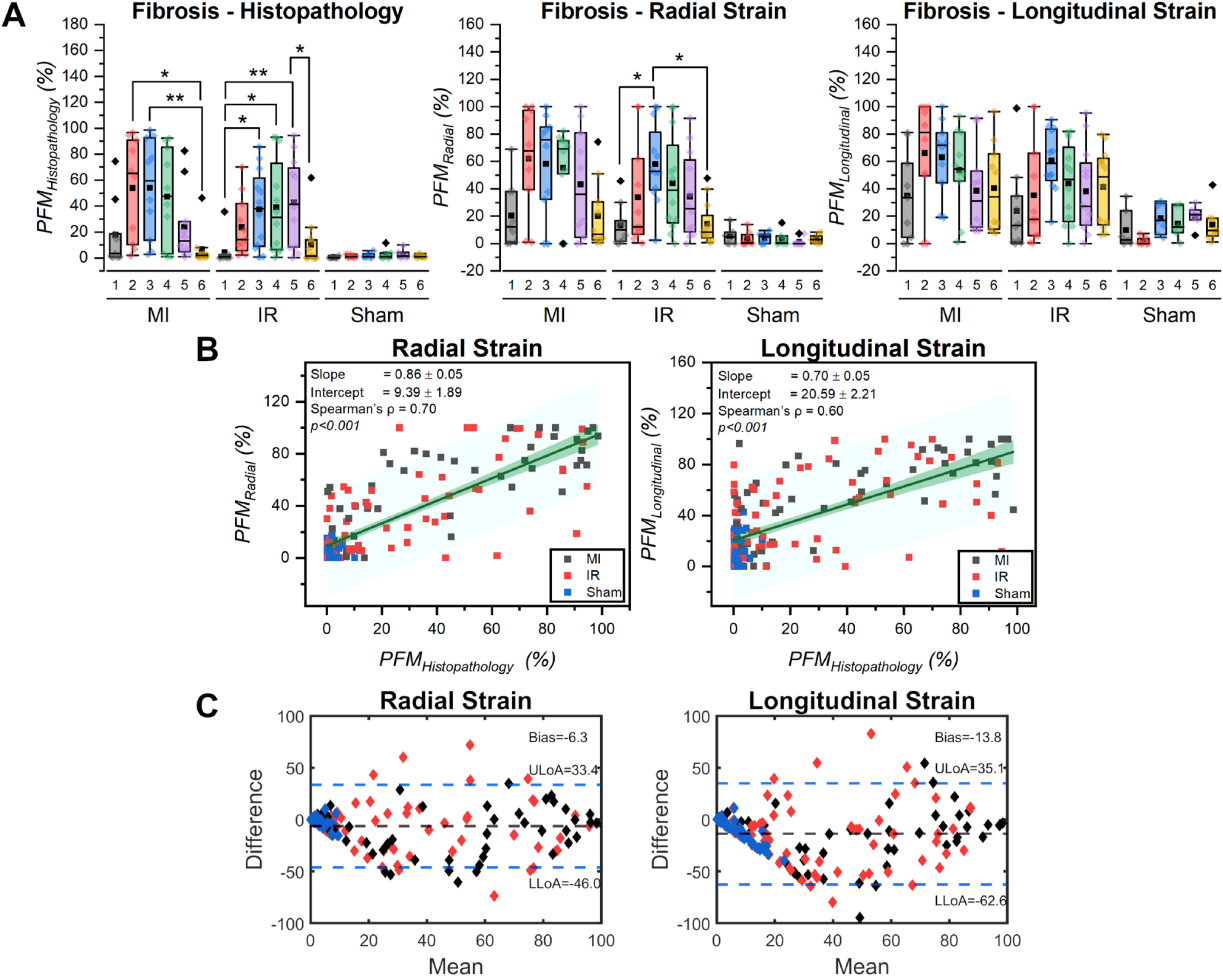
Quantitative comparison between cardiac fibrosis maps estimated using strain images in vivo and WSI data ex vivo*.* (**A**) *From left to right:* Box and whisker plots demonstrating regional variation and inter-group comparison [MI (n = 10), IR (n = 13) and Sham (n = 8)] of PFM estimated from histopathology WSI data (*PFM*_*Histopathology*_), radial strain images (*PFM*_*Radial*_) and longitudinal strain images (*PFM*_*Longitudinal*_) respectively. Symbol definitions: 1—Anterior Base, 2—Anterior Mid, 3—Anterior Apex, 4—Posterior Apex, 5—Posterior Mid, 6—Posterior Base, Squares—mean, Diamonds—outliers, whiskers—1.5 × IQR (Inter-quartile range), box—25th to 75th percentiles. Kruskal–Wallis test with Bonferroni–Dunn test post hoc test was used. (**B**) *Left panel:* Correlation and linear regression analysis of *PFM*_*Radial*_ against *PFM*_*Histopathology*_ (n = 172). *Right panel:* Correlation and linear regression analysis of *PFM*_*Longitudinal*_ against *PFM*_*Histopathology*_ (n = 172). Linear regression slope and intercept, Pearson’s correlation coefficient and *p-*values are shown in upper left corner of each figure. (**C**) *From left to right:* Bland–Altman analyses for *PFM*_*Radial*_ and *PFM*_*Longitudinal*_ results, respectively. Difference axes denote Histopathology PFM–Strain PFM (either radial or longitudinal). Black and blue dotted lines show the best estimate of bias and its limits of agreement (LoA) respectively. Symbol definitions: Blue diamonds—sham, Red diamonds—IR, Black diamonds—MI.

Quantitative analysis of histopathology slides demonstrates that the apical and mid segments (segments 2–5) were impacted more in the MI and IR groups (higher PFM_Histopathology_ (%) values in Fig. 5A, Kruskal–Wallis test *p* = 0.002 and 0.001 respectively) compared to basal segments (statistically significant variations shown in Fig. 5A with asterisks). The sham group had lower PFM_Histopathology_ (%) values compared to the MI and IR groups (Kruskal–Wallis test p < 0.001; Bonferroni–Dunn post test results for Sham vs IR, *p* < 0.001 and Sham vs MI, *p* < 0.001). A similar trend was observed in radial strain fibrosis results with relatively higher PFM_Radial_ (%) values for the MI and IR groups in the mid and apical segments compared to the basal segment. However, the inter segment variation was only significant for the IR group (Kruskal–Wallis test p = 0.005), and not for the MI group (Kruskal–Wallis test p = 0.182). The sham group also demonstrated lower PFM_Radial_ (%) values compared to the MI and IR groups (Kruskal–Wallis test p < 0.001; Bonferroni–Dunn post test results for Sham vs IR, *p* < 0.001 and Sham vs MI, *p* < 0.001). Longitudinal strain fibrosis results presented with higher standard deviation in the sham group when compared to radial strain measurements (Supplementary Table S19). The standard deviation values of PFM_Longitudinal_ were compared against corresponding values of PFM_Radial_ using independent samples Mann–Whitney *U* test and the results were found to be significantly different with a p-value of 0.041.

Both PFM_Radial_ and PFM_Longitudinal_ results demonstrated strong, positive correlation with PFM_Histopathology_ evaluated using Spearman’s rank correlation. However, PFM_Radial_ had a higher correlation value (*r* = 0.70, fitted line slope = 0.86, n = 172, *p* < 0.001) compared to PFM_Longitudinal_ (*r* = 0.60, fitted line slope = 0.70, n = 172, *p* < 0.001) considering all surgical groups (see Table 1). Similar trends of higher correlation with PFM_Radial_ were also observed when correlation analysis was done for individual injury groups (MI and IR in Table 1). Bland–Altman analysis (Fig. 5C) shows that both strain fibrosis measures overestimate the area of fibrosis with radial strain having lower bias (estimate of bias = − 6.3) compared to longitudinal strain (estimate of bias = − 13.8).

**Table 1:** Correlation of strain parameters against fibrotic myocardium measured with histopathology.

Surgical group	Strain parameter	Spearman’s *ρ* *r*-value	Confidence interval^a,b^		*p*-value
			Lower	Upper	
MI, IR, and Sham	PFM_Radial_	**0.70**	0.60	0.76	< 0.001
	PFM_Longitudinal_	0.60	0.49	0.69	< 0.001
MI	PFM_Radial_	**0.80**	0.67	0.88	< 0.001
	PFM_Longitudinal_	0.72	0.56	0.82	< 0.001
IR	PFM_Radial_	**0.64**	0.47	0.76	< 0.001
	PFM_Longitudinal_	0.46	0.24	0.63	< 0.001

^a^Estimation is based on Fisher's r-to-z transformation.

^b^Estimation of standard error is based on the formula proposed by Fieller, Hartley, and Pearson.

Highest correlation values for each surgical group's correlation analysis are shown in bold.

## Discussion

This study presents an adaptive Bayesian regularized cardiac strain imaging (ABR-CSI) algorithm for in vivo murine cardiac fibrosis localization (Fig. 1). ABR-CSI had been previously validated in FEA cardiac simulation^35^ along with limited in vivo evaluation in healthy murine hearts^31^. In this study, ABR-CSI was used to derive the PFM parameter as an in vivo surrogate measure of myocardial fibrosis in a larger cohort of murine injury models. Murine MI model enables investigation of long-term remodeling and is relevant in human patients not receiving timely reperfusion after acute ST segment elevation MI (STEMI)^36^. The IR model allows for investigation of adverse remodeling associated with reperfusion after acute STEMI^36^. Our results indicate that ABR-CSI can be utilized for the serial investigation of LV remodeling associated with these murine models (Figs. 2 and 3). Presented results also demonstrate strong association of the ABR-CSI PFM parameter with histopathology findings.

RF-based CSI methods are prone to motion estimation inaccuracies due to signal decorrelation associated with large deformation^22,25^, thus making accurate strain estimation in murine models challenging^37^. Luo et al*.* utilized a custom imaging sequence (EKV mode) for performing RF data collection at a higher frame rate to address the signal decorrelation problem. However, results were only limited to axial strain images with no histopathological validation^26^. Even though authors claimed that lateral motion was negligible in the PLAX views, previous studies showed statistically significantly different longitudinal strain values post-surgery (MI and IR)^10,21^. In contrast to previous methods, ABR-CSI provides accurate estimation of both radial and longitudinal strain in mice with more robust use of RF data. This is attributed to its ability to handle large deformation and dynamic variation of regularization under different degrees of signal decorrelation. Furthermore, the adaptive nature of the algorithm provides safeguards against excessive regularization which tends to reduce tracking resolution^31,38^. ABR-CSI is also a data driven approach in contrast to model based regularization methods^39^ thus eliminating regularization sensitivity to specific tuning parameters^31^.

Strain derived fibrosis maps presented in this paper enable concise visualization of fibrosis progression post-surgery associated with LV remodeling (Figs. 2A and 3A). ES strain images were utilized for fibrosis quantification as it is the recommended default time point for describing myocardial deformation from a recent strain imaging standardization task force^38^. Vincent et al*.* also corroborates our approach of using ES strain images^24^. Two separate threshold values had to be chosen for radial ($e_{r}^{th}$ = 4%) and longitudinal ($e_{l}^{th}$ = − 4%) strain images due to their opposing BL strain direction (myocardial thickening and shortening^10^ respectively). Threshold values were chosen based on maximizing the correlation between PFM_Radial_ and PFM_Longitudinal_ against PFM_Histopathology_ separately for this study. In the absence of ground truth histopathology images, BL data collected from a larger cohort of mice can be employed for choosing the threshold^24^. Quantitative analysis of fibrosis maps was performed regionally rather than globally due to the non-uniform LV structural and functional remodeling expected in MI and IR^6^. PFM_Radial_ values were relatively preserved in basal segments compared to mid and apical segments (Fig. 2B), while PFM_Longitudinal_ values were impaired considerably in all segments except the anterior base (Fig. 3B). A study using STE from Bhan et al*.* corroborate these findings where authors found well preserved radial strain in basal segments and significantly impaired longitudinal strain around the myocardium 4 weeks post-MI^21^. The authors posited that increased sensitivity of longitudinal strain in the basal segments might be related to alteration of endocardial fiber motion and interaction between hypertrophic and fibrotic regions^21^. Similarly, Bauer et al*.* also observed impaired STE-derived longitudinal strain in remote regions 1 week post-MI and claimed that these functional abnormalities might be related to global cardiac stunning and hypoperfusion^10^. However, our histopathological image analysis (Fig. 4 and Supplementary Fig. S1) demonstrates that basal segments with preserved PFM_Radial_ and impaired PFM_Longitudinal_ correspond to remote regions without any fibrosis thus indicating radial strain might be a better marker to differentiate between scar (fibrosis) and remote (non-fibrotic) regions.

To remove any user induced bias in fibrosis quantification from MT-strained histopathology WSI, an open source machine learning based classification tool (QuPath)^33^ was utilized (Fig. 1C). MT staining has previously been reported to reliably locate fibrosis as early as 72 h post ischemia^40^. For this study, our imaging end point was 2 weeks and therefore we used MT to identify areas of fibrosis on histopathology slides^40^. Due to spatial variation of fibrosis location in the MI and IR groups, correlation and linear regression analysis was also carried out regionally. Strong positive correlation of PFM_Radial_ and PFM_Longitudinal_ results with PFM_Histopathology_ indicate that ABR-CSI derived fibrosis parameters can reliably localize cardiac fibrosis in the myocardium. Correlation co-efficient values (Fig. 5B) were relatively higher compared to the studies reported previously in the literature^7,21^. Bhan et al*.* found *r* = 0.63 for radial strain and *r* = 0.74 for longitudinal strain in MI mice using parasternal long axis data^21^ against MRI-derived infarct size while Peng et al*.* reported *r* = − 0.32 for radial strain against cardiac fibrosis derived from MT-stained slides 4 weeks post MI^7^. It is worth noting that none of these studies considered spatial variation of fibrosis locations, instead focused on total percentage of infarcted myocardium. Overestimation of percentage PFM values observed in the Bland–Altman analysis (Fig. 5C) between in vivo strain and ex vivo WSI derived fibrosis might be attributed to the resolution difference between high frequency ultrasound (axial resolution = 22 µm) and WSI scanner (pixel mapping = 0.25 µm).

The findings of this study provide confidence that RF based CSI with adaptive Bayesian regularization has the potential to evaluate the efficacy of therapeutic intervention such as stem cell therapy to treat individuals with myocardial injury due to CHD^41,42^. The findings of this study also suggest that ABR-CSI has the potential to assess and identify segments of dysfunctional myocardium using the proposed PFM parameter. This work lays the foundation of translating ABR-CSI from the pre-clinical investigation to the clinical domain. Future work will focus on translating ABR-CSI to evaluate subtler myocardial ischemia situations associated with doxorubicin-induced cardiotoxicity or hypertensive murine models. Based on our results demonstrating the ability to detect smaller infarcts (Supplementary Fig. S1), we believe ABR-CSI induced strain parameters would be able to detect the expected subtle changes associated with these models.

Finally, there are several limitations of this study. First, 2-D ultrasound imaging planes and 2-D histopathology (selected from a 3-D stack) were manually selected and co-registered by an expert sonographer which may be a potential source of variability in the correlation analysis. Second, we observed some variability in tissue MT-stain color across the murine hearts potentially causing some misclassification with the random forest classifier used for fibrosis detection, however, the impact on our results was not significant. Third, to fully evaluate the extent of these injuries, 3-D CSI^43^ would be preferred instead of the 2-D approach reported here. Future studies will focus on bi-plane imaging with PLAX and parasternal short axis views^11^ or the implementation of ABR-CSI in 3-D.

## Conclusion

We have demonstrated the feasibility of an adaptive Bayesian regularized cardiac strain imaging (ABR-CSI) algorithm for in vivo murine myocardial function assessment. ABR-CSI was able to localize cardiac fibrosis using both radial and longitudinal strain images and demonstrated strong correlation against ground truth fibrosis derived from histopathological image analysis.

## Materials and methods

### Murine models and surgical procedures

All in vivo experimental protocols were approved by the University of Wisconsin (UW)-Madison, Institutional Research Animal Resources and Compliance (RARC) Committee and all experimental methods were conducted according to the approved guidelines from RARC, UW-Madison. All relevant methods are reported following recommendations from ARRIVE (Animal Research: Reporting of In Vivo Experiments) guidelines^44^. A total of 43 healthy BALB/CJ mice (median age of 10 weeks, 21 males [weight (g): mean = 26.86, range = 6.40], 22 females [weight (g): mean = 20.72, range = 5.60]) acquired from The Jackson Laboratory (Bar Harbor, ME USA) were evaluated in cohorts of 3 (one MI, one IR and one Sham) at pre-surgery (BL) and 1, 2, 7 and 14 days post-surgery. For surgical procedures, anesthesia was induced with 3–5% isoflurane. Mice were intubated and placed on a ventilator at 175 breaths/min and 200–300 µl tidal volume/breath. Isoflurane was introduced through the ventilator at 1.5–2.0% and maintained for the duration of the procedure. A left lateral incision was made in the fourth intercostal space to allow access to the heart. 7-0 prolene suture was placed through the myocardium in the anterolateral wall. The suture was secured in place ligating the left anterior descending artery (LAD) and MI was confirmed by observing blanching of the distal circulation (ventricular apex)^45^. For the IR group, the LAD suture was removed after 45 min. The ribs and muscle layers were closed by 6-0 absorbable suture while evacuating the chest. The skin was closed using 6-0 non-absorbable suture. The mouse was then recovered from anesthesia and extubated. The sham group underwent similar procedures with no manipulation of the heart. 12 mice did not survive the surgical procedure resulting in a final sample size of 31 (gender: 17 females, 14 males, surgical group: MI = 10, IR = 13, Sham = 8) for the longitudinal study.

### Ultrasound radio-frequency (RF) data collection

A Vevo 2100 ultrasonic imaging system with a MS-550D transducer (FUJIFILM VisualSonics, Inc., Toronto, Canada) was utilized for collecting simultaneous B-mode and RF data. Mice were placed in an induction chamber of 3–5% isoflurane at a rate of 1–2 L/min of oxygen. For the initial imaging session (pre-surgery), chest hair was removed with depilatory cream, Nair (Church & Dwight Co., Ewing, NJ), and the mice were transferred to a heated platform and placed in the supine position. Anesthesia was maintained via nosecone at 1.5–3.5% isoflurane and a continuous flow of oxygen (1–2 L/min). ECG and respiratory signals were collected using a dedicated physiological monitoring system included with the Vevo 2100. Spectra 360 electrode gel (Parker Labs, Fairfield, NJ) was applied on the physiological signal monitoring system electrodes to ensure optimal contact with each paw ensuring high-quality ECG and respiratory signal acquisition. The supply of isoflurane and oxygen flow rate was titrated to maintain a consistent heart rate between 310–340 beats per minute (bpm) as best as possible during all image acquisition sessions. Using a MS-550D transducer (f_c_ = 30 MHz), 1000 frames of RF data at 235 fps in parasternal long axis (PLAX) view were acquired. To ensure reproducibility over serial investigations, a clear view of the left ventricular outflow tract (LVOT) was used as an anatomical landmark. Finally, collected RF data were exported using VevoLab software in IQ (in-phase and quadrature) format for offline cardiac strain estimation.

### Adaptive Bayesian regularized cardiac strain imaging (ABR-CSI)

Cardiac strain estimation was performed using RF data encompassing one cardiac cycle extracted by ECG and respiratory signal gating. First, inter-frame axial and lateral displacements were estimated using a multi-level block matching algorithm with adaptive Bayesian regularization (ABR)^31^. ABR applies Bayesian regularization^29,46^ iteratively on 2-D normalized cross-correlation (2-D NCC) functions to incorporate spatial smoothness using information from adjacent neighbors. Local signal decorrelation and signal-to-noise ratio of 2-D NCC functions determine the optimal number of iterations in the ABR algorithm. ABR is discussed in detail elsewhere^31^. Displacement estimation parameters are listed in Table 2.

**Table 2:** Displacement estimation parameters.

Parameters	Value	Unit
Number of levels	3	–
RF data sampling factor [axial:lateral]	1:2	–
Axial decimation factors	[3,2,1]	–
Lateral decimation factors	[2,1,1]	–
Axial kernel length	[$8\lambda$, $5\lambda$, $1\lambda$]	Wavelengths
Lateral kernel length	[15, 12, 10]	A-lines
Kernel overlaps [axial, lateral]	[50, 90]	%
Axial displacement median filter kernel	[0.13 × 0.41]	mm
Lateral displacement median filter kernel	[0.30 × 0.90]	mm
Subsample estimation method	2-D Sinc^47,48^	–
Least squares kernel [axial, lateral]	[0.06, 0.5]	mm
Maximum ABR iteration	10	–

Next, a mesh of 24,000 points (40 × 600 points in radial and circumferential orientation respectively) covering the entire myocardium was generated by manually segmenting epicardial and endocardial walls at end-diastole (R-Wave of ECG). The generated cardiac mesh was utilized to accumulate incremental displacements over the cardiac cycle. The Lagrangian strain tensor containing axial, lateral and shear strain components was calculated using a least squares strain estimator on the accumulated displacement fields. Finally, cartesian to cardiac coordinate transformation was applied on the Lagrangian strain tensor to estimate radial and longitudinal strains. Further details on the accumulation and strain estimation process can be found here^29^.

ES radial and longitudinal strain images were selected corresponding to the cardiac phase with smallest LV chamber area^7^. Cardiac fibrosis maps were generated by applying a binary thresholding operation on the ES strain images with strain thresholds set to $e_{r}^{th}$ = 4% for radial and $e_{l}^{th}$ = − 4% for longitudinal. For radial strain, pixels less than $e_{r}^{th}$ were assigned as fibrotic while for longitudinal, pixels greater than $e_{l}^{th}$ were assigned to the fibrotic class. Segmental analysis was performed by dividing the myocardium into six equal segments following the American Heart Association recommendation^34^. For each segment, percentage fibrotic myocardium (PFM)^24^ was calculated as an in vivo surrogate measurement of cardiac fibrosis using the following equation.

$$ PFM_{Strain} \;\left( \% \right) = \frac{Number \;of \;fibrotic \;pixels}{{Total \;number\; of\; pixels}} \times 100 $$

Segments corrupted by signal dropouts from ribs or sutures^37^, reverberation signals from the chest wall, ribs or sternum^49^ and acoustic shadowing^5^ were excluded from statistical analysis.

### Histopathological data analysis

After the 14th day post-surgery imaging session, mice were euthanized via CO_2_ asphyxiation. Hearts were collected for histology, rinsed with Phosphate Buffered Saline and rinsed again with 10% Neutral Buffered Formalin (NBF) to ensure chamber inflation. Tissues were then placed in 10% NBF for a minimum of 24 h. Post-fixation, Tissue Marking Dye (Davidson Marking System, Bradley Products, Inc., New Jersey, USA) was applied to the anterior surface of each heart in two planes: the first indicating the approximate location of the aortic outflow tract, and the second perpendicular to this. Hearts were placed in tissue cassettes and underwent standard formalin to paraffin tissue processing on a Tissue-Tek VIP processor (Sakura Finetek, USA). Harvested myocardial tissues were embedded in paraffin highlighting the aortic outflow tract to achieve an orientation roughly approximating the ultrasonographic PLAX plane. 5 µm-thick sections were taken at 100 µm intervals through the entire heart in the long axis orientation and stained with Masson’s trichome (MT) for quantification of fibrosis. The stained slides were digitized using a 20 × uScopeHXII digital microscope (Microscopes International, Dallas, TX). 2-D ultrasound imaging planes and histopathology tissue sections were manually selected and co-registered by an expert sonographer. To automatically classify regions of fibrosis in the digital WSI, a 4-class Random Tree pixel classifier was designed using QuPath^33^ (an open-source software for digital pathology image analysis accessed through: https://qupath.github.io/) by manually delineating fibrotic (red), non-fibrotic (green), blood clots (blue) and background (black) regions in six representative WSIs. Note that, fibrotic (collagen) and non-fibrotic regions were stained as blue and dark purple in the MT stained WSIs. Finally, classified images from QuPath^33^ were loaded into MATLAB (R2018b, The MathWorks, Inc., Natick, MA, USA) to quantify percentage of fibrosis in the infarct and viable regions. To perform the segmental analysis, classified WSIs were divided into six segments like the in vivo ultrasound images and PFM values were calculated per segment using the following equation.

$$ PFM_{Histopathology} \;\left( \% \right) = \frac{Fibrotic \;Pixels}{{Total \;Pixels - Background \;Pixels}} \times 100 $$

One IR mouse had to be excluded from the final analysis due to poor MT stain quality.

### Statistical analysis

The results are presented as mean ± standard error (SE) of the mean unless otherwise stated. Since our data is not well-modeled by a normal distribution assessed using the Shapiro–Wilk test and small sample size in each surgical group (Sham = 8, MI = 10, IR = 13 with occasional smaller sample sizes due to data rejection associated with ultrasound signal corruption), non-parametric statistical analysis was employed to determine statistical significance in this study. Friedman’s test with Bonferroni–Dunn^50,51^ post hoc test was used to evaluate the longitudinal variation in the strain measures over time (repeated measurements) within the surgical groups (MI, IR and Sham). Bonferroni–Dunn test was chosen for its adequate control of Type I error through Bonferroni correction for multiple pairwise comparisons^50,52^. To evaluate differences among surgical groups (Sham versus MI, MI versus IR, IR versus Sham) at a specific imaging time point, Kruskal–Wallis one-way analysis of variance on ranks with Bonferroni–Dunn^50,51^ post hoc test was used. Kruskal–Wallis test with Bonferroni–Dunn test^50,51^ post hoc test was also used to evaluate inter-segment variation of cardiac fibrosis estimated from WSI and strain imaging data at the final imaging point. Associations between strain and histopathology derived fibrosis measures were evaluated using Spearman’s rank correlation, and the agreements were presented using Bland–Altman plots. Linear fitting was done to access the trend between strain and histopathology derived fibrosis measures. A two-sided p-value < 0.05 was considered to be statistically significant. All the statistical analyses were performed using IBM SPSS Statistics (IBM Corp. Released 2021. IBM SPSS Statistics for Windows, Version 28.0. Armonk, NY: IBM Corp). Results were plotted using Origin (Version 2020, OriginLab Corporation, Northampton, MA, USA) and MATLAB (R2018b, The MathWorks, Inc., Natick, MA, USA).

## Supplementary Information

Supplementary Information.

## Data Availability

All relevant data is presented in the paper.
