# Supplementary material for: Murine cardiac fibrosis localization using adaptive Bayesian cardiac strain imaging in vivo
Source: Sci Rep. 2022 May 20;12:8522. doi: 10.1038/s41598-022-12579-6 (PMC9122999; doi:10.1038/s41598-022-12579-6)
Supplement: Supplementary file 1 — Supplementary Information. [file 41598_2022_12579_MOESM1_ESM.pdf]

Murine Cardiac Fibrosis Localization using Adaptive Bayesian Cardiac Strain Imaging *In Vivo*

Rashid Al Mukaddim[1]*, Ashley M. Weichmann[2], Rachel Taylor[3], Timothy A. Hacker[3], Thomas Pier[4], Joseph Hardin[4], Melissa Graham[5], Carol C. Mitchell[6] and Tomy Varghese[1*]

[1]Medical Physics, University of Wisconsin (UW) – Madison

[2]Small Animal Imaging and Radiotherapy Facility, UW-Madison

[3]Cardiovascular Physiology Core Facility, UW-Madison

[4]Experimental Animal Pathology Lab, UW-Madison

[5]Comparative Pathology Laboratory, Research Animal Resources and Compliance (RARC), UW-Madison

[6]Medicine/Division of Cardiovascular Medicine, UW-Madison

*corresponding author email – mukaddim@wisc.edu, tvarghese@wisc.edu

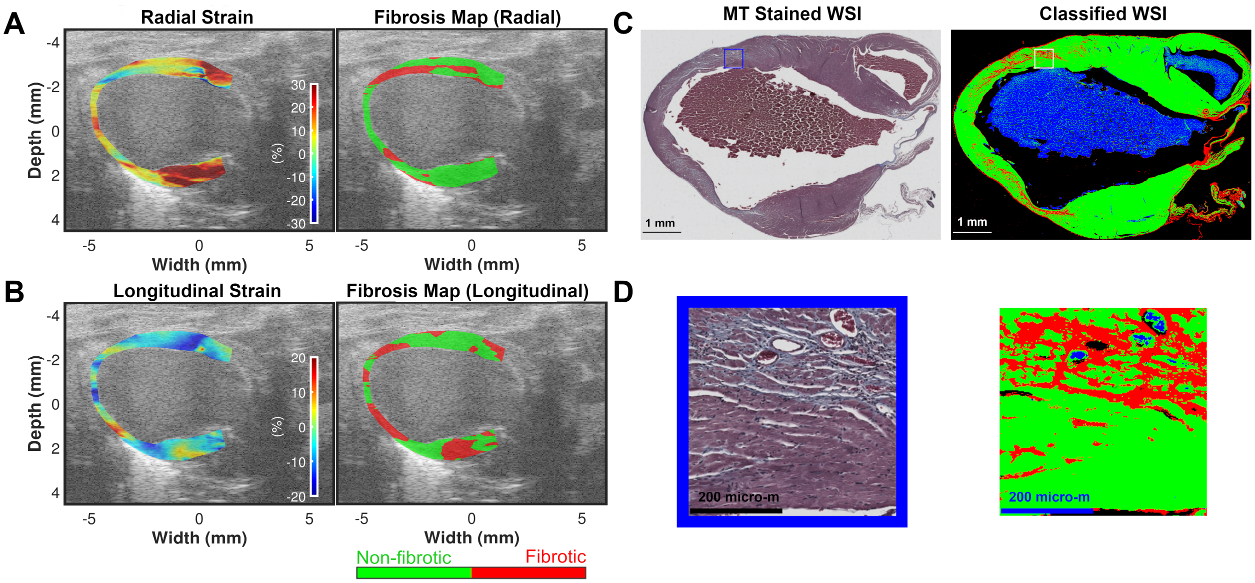

Supplementary Figure S1. Qualitative results showing capability of strain fibrosis maps for detecting smaller MI in a male BALB/CJ mouse after ischemia-reperfusion surgery. (A) Left panel: Cumulative ES radial strain image at Day 14. Right panel: Corresponding cardiac fibrosis map. **(B)** Cumulative ES longitudinal strain image at Day 14. Right panel: Corresponding cardiac fibrosis map. Red = fibrotic region, green = non-fibrotic region. **(C)** Left panel: MT stained WSI. Right panel: Corresponding classified image generated using a random forest classifier. Red = fibrotic region, green = non-fibrotic region, blue = blood clot, black = background. **(D)** Left panel: A magnified region-of-interest extracted from the WSI image demonstrating collagen deposition in the fibrotic regions. Right panel: Corresponding classified image using the random forest classifier.

Temporal Variation of Strain Fibrosis Parameters Straited by Surgical Groups

This section presents results using Friedman's test with Bonferroni-Dunn post hoc test used to evaluate the longitudinal variation of strain measures over time (repeated measurements) within the surgical groups (myocardial infarction [MI], ischemia-reperfusion [IR] and Sham). Tables S1 to S3 denote PFM_{Radial} results for IR, MI and Sham group respectively. Tables S4 to S6 denote the $PFM_{Longitudinal}$ results for IR, MI and Sham group respectively.

Table S1: Surgical Group = Ischemia Reperfusion, Data = PFM_{Radial}

Segment	Friedman's Test *p* (chi-square statics, DOF)	Multiple Comparison	Bonferroni-Dunn Test *p*
01 (n = 7)	**0.03 (10.52, 4)**	BL - D1	1.00
		BL - D2	1.00
		BL- D7	0.430
		BL - D14	0.280
02 (n = 8)	**0.0003 (20.56, 4)**	BL - D1	**0.002**
		BL - D2	1.00
		BL- D7	**0.044**
		BL - D14	**0.004**
03 (n =8)	**0.027 (10.94, 4)**	BL - D1	0.114
		BL - D2	0.690
		BL- D7	0.398
		BL - D14	**0.02**
04 (n = 6)	**0.012 (12.93, 4)**	BL - D1	0.062
		BL - D2	1.00
		BL- D7	**0.010**
		BL - D14	0.176
05 (n = 9)	**0.013 (12.71, 4)**	BL - D1	0.171
		BL - D2	0.253
		BL- D7	**0.006**
		BL - D14	1.000
06 (n = 9)	0.071 (8.62, 4)	BL - D1	Non sig Friedman's Test
		BL - D2	
		BL- D7	
		BL - D14	

Table S2: Surgical Group = Myocardial Infarction, Data = PFM_{Radial}

Segment	Friedman's Test *p* (chi-square statics, DOF)	Multiple Comparison	Bonferroni-Dunn Test *p*
01 (n = 6)	0.109 (7.56, 4)	BL - D1	Non sig Friedman's Test
		BL - D2	
		BL- D7	
		BL - D14	
02 (n = 9)	**0.0004 (20.33, 4)**	BL - D1	0.139
		BL - D2	1.00
		BL- D7	**0.01**
		BL - D14	**0.006**

03 (n = 9)	**0.013 (15.58, 4)**	BL - D1	**0.014**
		BL - D2	0.624
		BL- D7	**0.046**
		BL - D14	0.736
04 (n = 9)	**0.006 (14.64, 4)**	BL - D1	**0.037**
		BL - D2	**0.014**
		BL- D7	**0.017**
		BL - D14	0.526
05 (n = 10)	**0.014 (12.56, 4)**	BL - D1	**0.019**
		BL - D2	0.196
		BL- D7	**0.030**
		BL - D14	0.284
06 (n = 10)	**0.033 (10.48, 4)**	BL - D1	0.162
		BL - D2	1.00
		BL- D7	1.00
		BL - D14	0.237

Table S3: Surgical Group = Sham, Data = PFM_{Radial}

**Segment**	**Friedman's Test *p* (chi-square statics, DOF)**	**Multiple Comparison**	**Bonferroni-Dunn Test *p***
01 (n = 4)	0.95 (0.71, 4)	BL - D1	Non sig Friedman's Test
		BL - D2	
		BL- D7	
		BL - D14	
02 (n = 7)	0.237 (5.53, 4)	BL - D1	Non sig Friedman's Test
		BL - D2	
		BL- D7	
		BL - D14	
03 (n = 5)	0.282 (5.05, 4)	BL - D1	Non sig Friedman's Test
		BL - D2	
		BL- D7	
		BL - D14	
04 (n = 5)	0.315 (4.74, 4)	BL - D1	Non sig Friedman's Test
		BL - D2	
		BL- D7	
		BL - D14	
05 (n = 7)	0.098 (7.82, 4)	BL - D1	Non sig Friedman's Test
		BL - D2	
		BL- D7	
		BL - D14	
06 (n = 8)	0.784 (1.74, 4)	BL - D1	Non sig Friedman's Test
		BL - D2	
		BL- D7	
		BL - D14	

Table S4: Surgical Group = Ischemia Reperfusion, Data = $PFM_{Longitudinal}$

**Segment**	**Friedman's Test *p* (chi-square statics, DOF)**	**Multiple Comparison**	**Bonferroni-Dunn Test *p***
01 (n = 7)	0.136 (6.99, 4)	BL - D1	

		BL - D2	Non sig Friedman's Test
		BL- D7	
		BL - D14	
02 (n = 8)	**0.002 (17.01, 4)**	BL - D1	**0.003**
		BL - D2	0.219
		BL- D7	**0.009**
		BL - D14	0.091
03 (n = 8)	0.081 (8.30, 4)	BL - D1	Non sig Friedman's Test
		BL - D2	
		BL- D7	
		BL - D14	
04 (n = 6)	0.048 (9.60, 4)	BL - D1	0.106
		BL - D2	1.00
		BL- D7	0.106
		BL - D14	1.00
05 (n = 9)	0.224 (5.68, 4)	BL - D1	Non sig Friedman's Test
		BL - D2	
		BL- D7	
		BL - D14	
06 (n =9)	0.036 (10.31, 9)	BL - D1	1.00
		BL - D2	1.00
		BL- D7	0.073
		BL - D14	0.369

Table S5: Surgical Group = Myocardial Infarction, Data = $PFM_{Longitudinal}$

Segment	Friedman's Test *p* (chi-square statics, DOF)	Multiple Comparison	Bonferroni-Dunn Test *p*
01 (n = 6)	0.076 (8.47, 4)	BL - D1	Non sig Friedman's Test
		BL - D2	
		BL- D7	
		BL - D14	
02 (n = 9)	**0.0004 (20.39, 4)**	BL - D1	0.209
		BL - D2	1.000
		BL- D7	**0.006**
		BL - D14	**0.006**
03 (n = 9)	**0.004 (15.37, 4)**	BL - D1	0.171
		BL - D2	0.253
		BL- D7	**0.001**
		BL - D14	0.526
04 (n = 9)	**0.05 (9.51, 4)**	BL - D1	**0.046**
		BL - D2	0.369
		BL- D7	0.171
		BL - D14	1.000
05 (n = 10)	**0.006 (14.32, 4)**	BL - D1	**0.047**
		BL - D2	**0.011**
		BL- D7	0.339
		BL - D14	1.00
06 (n =10)	**0.003 (15.76, 4)**	BL - D1	0.109
		BL - D2	0.477
		BL- D7	**0.004**
		BL - D14	**0.011**

Table S6: Surgical Group = Sham, Data = $PFM_{Longitudinal}$

**Segment**	**Friedman's Test *p* (chi-square statics, DOF)**	**Multiple Comparison**	**Bonferroni-Dunn Test *p***
01 (n = 4)	0.975 (0.48, 4)	BL - D1	Non sig Friedman's Test
		BL - D2	
		BL- D7	
		BL - D14	
02 (n = 7)	0.312 (4.77, 4)	BL - D1	Non sig Friedman's Test
		BL - D2	
		BL- D7	
		BL - D14	
03 (n = 5)	0.205 (5.920,4)	BL - D1	Non sig Friedman's Test
		BL - D2	
		BL- D7	
		BL - D14	
04 (n = 5)	0.634 (2.56, 4)	BL - D1	Non sig Friedman's Test
		BL - D2	
		BL- D7	
		BL - D14	
05 (n = 7)	0.869 (1.25, 4)	BL - D1	Non sig Friedman's Test
		BL - D2	
		BL- D7	
		BL - D14	
06 (n = 8)	0.039 (10.10, 4)	BL - D1	0.072
		BL - D2	0.114
		BL- D7	0.114
		BL - D14	0.820

Intergroup Comparison Straited by Imaging Time Points Per Myocardial Segments

This section presents the results for Kruskal–Wallis one-way analysis of variance on ranks with Bonferroni-Dunn[1,2] post hoc test was done per segment to evaluate differences among surgical groups (Sham versus MI, MI versus IR, IR versus Sham) at a specific imaging time point. Tables S7 to S12 denote the PFM_{Radial} results for segments 1 - 6, respectively. Tables S13 to S18 denote the $PFM_{Longitudinal}$ results for segments 1 - 6, respectively. Segment definitions: 1 – Anterior Base, 2 – Anterior Mid, 3 – Anterior Apex, 4 – Posterior Apex, 5 – Posterior Mid, 6 – Posterior Base.

Table S7: Segment = 1, Data = PFM_{Radial}

Imaging Time Point (n)	Kruskal-Wallis Test *p* (chi-square statics, DOF)	Multiple Comparison	Bonferroni-Dunn Test *p*
Baseline (n = 23)	0.197 (3.25, 2)	Sham-IR	Non sig Kruskal-Wallis Test
		Sham-MI	
		IR-MI	
Day 1 (n = 27)	0.380 (1.93, 2)	Sham-IR	Non sig Kruskal-Wallis Test
		Sham-MI	
		IR-MI	
Day 2 (n = 23)	0.364 (2.021, 2)	Sham-IR	Non sig Kruskal-Wallis Test
		Sham-MI	
		IR-MI	
Day 7 (n = 23)	0.836 (0.358, 2)	Sham-IR	Non sig Kruskal-Wallis Test
		Sham-MI	
		IR-MI	
Day 14 (n = 24)	0.148 (3.817, 2)	Sham-IR	Non sig Kruskal-Wallis Test
		Sham-MI	
		IR-MI	

Table S8: Segment = 2, Data = PFM_{Radial}

Imaging Time Point (n)	Kruskal-Wallis Test *p* (chi-square statics, DOF)	Multiple Comparison	Bonferroni-Dunn Test *p*
Baseline (n =28)	0.133 (4.03, 2)	Sham-IR	Non sig Kruskal-Wallis Test
		Sham-MI	
		IR-MI	
Day 1 (n = 30)	0.083 (4.98, 2)	Sham-IR	Non sig Kruskal-Wallis Test
		Sham-MI	
		IR-MI	
Day 2 (n = 30)	0.692 (0.73, 2)	Sham-IR	Non sig Kruskal-Wallis Test
		Sham-MI	
		IR-MI	
Day 7 (n = 27)	**0.156 (3.71, 2)**	Sham-IR	Non sig Kruskal-Wallis Test
		Sham-MI	
		IR-MI	
Day 14 (n = 26)	**0.006 (10.35, 2)**	Sham-IR	0.078
		Sham-MI	**0.005**
		IR-MI	1.000

Table S9: Segment = 3, Data = PFM_{Radial}

Imaging Time Point (n)	Kruskal-Wallis Test *p* (chi-square statics, DOF)	Multiple Comparison	Bonferroni-Dunn Test *p*
Baseline (n = 25)	0.196 (3.26, 2)	Sham-IR	Non sig Kruskal-Wallis Test
		Sham-MI	
		IR-MI	
Day 1 (n = 31)	0.056 (5.78, 2)	Sham-IR	Non sig Kruskal-Wallis Test
		Sham-MI	
		IR-MI	
Day 2 (n = 31)	0.052 (5.90, 2)	Sham-IR	Non sig Kruskal-Wallis Test
		Sham-MI	
		IR-MI	
Day 7 (n = 27)	**0.006 (10.32, 2)**	Sham-IR	**0.025**
		Sham-MI	**0.007**
		IR-MI	1.00
Day 14 (n = 26)	**0.012 (8.79, 2)**	Sham-IR	**0.015**
		Sham-MI	**0.048**
		IR-MI	1.00

Table S10: Segment = 4, Data = PFM_{Radial}

Imaging Time Point (n)	Kruskal-Wallis Test *p* (chi-square statics, DOF)	Multiple Comparison	Bonferroni-Dunn Test *p*
Baseline (n = 25)	0.841 (0.345, 2)	Sham-IR	Non sig Kruskal-Wallis Test
		Sham-MI	
		IR-MI	
Day 1 (n = 31)	0.182 (3.40, 2)	Sham-IR	Non sig Kruskal-Wallis Test
		Sham-MI	
		IR-MI	
Day 2 (n = 31)	**0.008 (9.63, 2)**	Sham-IR	0.222
		Sham-MI	**0.006**
		IR-MI	0.336
Day 7 (n = 28)	**0.007 (9.94, 2)**	Sham-IR	**0.010**
		Sham-MI	**0.030**
		IR-MI	1.00
Day 14 (n = 26)	**0.016 (8.26, 2)**	Sham-IR	**0.041**
		Sham-MI	**0.026**
		IR-MI	1.00

Table S11: Segment = 5, Data = PFM_{Radial}

Imaging Time Point (n)	Kruskal-Wallis Test *p* (chi-square statics, DOF)	Multiple Comparison	Bonferroni-Dunn Test *p*
Baseline (n = 31)	0.360 (2.045, 2)	Sham-IR	Non sig Kruskal-Wallis Test
		Sham-MI	
		IR-MI	
Day 1	**0.001 (13.36, 2)**	Sham-IR	**0.006**

(n = 31)		Sham-MI	**0.002**
		IR-MI	1.00
Day 2<br>(n = 31)	0.063 (5.52, 2)	Sham-IR	Non sig Kruskal-Wallis Test
		Sham-MI	
		IR-MI	
Day 7<br>(n = 28)	**0.006 (10.40, 2)**	Sham-IR	**0.008**
		Sham-MI	**0.023**
		IR-MI	1.00
Day 14<br>(n = 26)	**0.002 (12.28, 2)**	Sham-IR	**0.006**
		Sham-MI	**0.005**
		IR-MI	1.00

Table S12: Segment = 6, Data = PFM_{Radial}

Imaging Time Point (n)	Kruskal-Wallis Test *p* (chi-square statics, DOF)	Multiple Comparison	Bonferroni-Dunn Test *p*
Baseline<br>(n = 31)	0.637 (0.90, 2)	Sham-IR	Non sig Kruskal-Wallis Test
		Sham-MI	
		IR-MI	
Day 1<br>(n = 31)	0.097 (4.66, 2)	Sham-IR	Non sig Kruskal-Wallis Test
		Sham-MI	
		IR-MI	
Day 2<br>(n = 31)	0.081 (5.03, 2)	Sham-IR	Non sig Kruskal-Wallis Test
		Sham-MI	
		IR-MI	
Day 7<br>(n = 28)	0.322 (2.26, 2)	Sham-IR	Non sig Kruskal-Wallis Test
		Sham-MI	
		IR-MI	
Day 14<br>(n = 27)	0.080 (5.061, 2)	Sham-IR	Non sig Kruskal-Wallis Test
		Sham-MI	
		IR-MI	

Table S13: Segment = 1, Data = $PFM_{Longitudinal}$

Imaging Time Point (n)	Kruskal-Wallis Test *p* (chi-square statics, DOF)	Multiple Comparison	Bonferroni-Dunn Test *p*
Baseline<br>(n = 23)	0.753 (0.57, 2)	Sham-IR	Non sig Kruskal-Wallis Test
		Sham-MI	
		IR-MI	
Day 1<br>(n = 27)	0.249 (2.77, 2)	Sham-IR	Non sig Kruskal-Wallis Test
		Sham-MI	
		IR-MI	
Day 2<br>(n = 23)	0.199 (3.234, 2)	Sham-IR	Non sig Kruskal-Wallis Test
		Sham-MI	
		IR-MI	
Day 7<br>(n = 23)	0.697 (0.72, 2)	Sham-IR	Non sig Kruskal-Wallis Test
		Sham-MI	
		IR-MI	
Day 14<br>(n = 24)	0.306 (2.37, 2)	Sham-IR	Non sig Kruskal-Wallis Test
		Sham-MI	
		IR-MI	

Table S14: Segment = 2, Data = $PFM_{Longitudinal}$

Imaging Time Point (n)	Kruskal-Wallis Test *p* (chi-square statics, DOF)	Multiple Comparison	Bonferroni-Dunn Test *p*
Baseline (n = 28)	0.272 (2.61, 2)	Sham-IR	Non sig Kruskal-Wallis Test
		Sham-MI	
		IR-MI	
Day 1 (n = 30)	0.118 (4.27, 2)	Sham-IR	Non sig Kruskal-Wallis Test
		Sham-MI	
		IR-MI	
Day 2 (n = 30)	0.110 (4.41, 2)	Sham-IR	Non sig Kruskal-Wallis Test
		Sham-MI	
		IR-MI	
Day 7 (n = 27)	**0.014 (8.60, 2)**	Sham-IR	0.128
		Sham-MI	**0.012**
		IR-MI	1.00
Day 14 (n = 26)	**0.005 (10.51, 2)**	Sham-IR	**0.052**
		Sham-MI	**0.005**
		IR-MI	1.000

Table S15: Segment = 3, Data = $PFM_{Longitudinal}$

Imaging Time Point (n)	Kruskal-Wallis Test *p* (chi-square statics, DOF)	Multiple Comparison	Bonferroni-Dunn Test *p*
Baseline (n = 25)	0.105 (4.51, 2)	Sham-IR	Non sig Kruskal-Wallis Test
		Sham-MI	
		IR-MI	
Day 1 (n = 31)	0.129 (4.10, 2)	Sham-IR	Non sig Kruskal-Wallis Test
		Sham-MI	
		IR-MI	
Day 2 (n = 31)	**0.032 (6.87, 2)**	Sham-IR	**0.045**
		Sham-MI	0.080
		IR-MI	1.00
Day 7 (n = 27)	**0.012 (8.82, 2)**	Sham-IR	0.077
		Sham-MI	**0.011**
		IR-MI	1.00
Day 14 (n = 26)	**0.005 (10.67, 2)**	Sham-IR	**0.014**
		Sham-MI	**0.009**
		IR-MI	1.000

Table S16: Segment = 4, Data = $PFM_{Longitudinal}$

Imaging Time Point (n)	Kruskal-Wallis Test *p* (chi-square statics, DOF)	Multiple Comparison	Bonferroni-Dunn Test *p*
Baseline (n = 25)	0.248 (2.79, 2)	Sham-IR	Non sig Kruskal-Wallis Test
		Sham-MI	
		IR-MI	
Day 1 (n = 31)	0.003 (11.88, 2)	Sham-IR	**0.008**
		Sham-MI	**0.005**
		IR-MI	1.00
Day 2 (n = 31)	0.006 (10.11, 2)	Sham-IR	**0.041**
		Sham-MI	**0.006**
		IR-MI	1.00
Day 7 (n = 28)	0.008 (9.63, 2)	Sham-IR	**0.021**
		Sham-MI	**0.017**
		IR-MI	1.00
Day 14 (n = 26)	0.044 (6.25, 2)	Sham-IR	0.159
		Sham-MI	**0.048**
		IR-MI	1.00

Table S17: Segment = 5, Data = $PFM_{Longitudinal}$

Imaging Time Point (n)	Kruskal-Wallis Test *p* (chi-square statics, DOF)	Multiple Comparison	Bonferroni-Dunn Test *p*
Baseline (n = 31)	0.795 (0.46, 2)	Sham-IR	Non sig Kruskal-Wallis Test
		Sham-MI	
		IR-MI	
Day 1 (n = 31)	0.096 (4.69, 2)	Sham-IR	Non sig Kruskal-Wallis Test
		Sham-MI	
		IR-MI	
Day 2 (n = 31)	**0.039 (6.49, 2)**	Sham-IR	**0.087**
		Sham-MI	**0.058**
		IR-MI	1.00
Day 7 (n = 28)	0.06 (5.1, 2)	Sham-IR	Non sig Kruskal-Wallis Test
		Sham-MI	
		IR-MI	
Day 14 (n = 26)	0.822 (0.392, 2)	Sham-IR	Non sig Kruskal-Wallis Test
		Sham-MI	
		IR-MI	

Table S18: Segment = 6, Data = $PFM_{Longitudinal}$

**Imaging Time Point (n)**	**Kruskal-Wallis Test *p* (chi-square statics, DOF)**	**Multiple Comparison**	**Bonferroni-Dunn Test *p***
Baseline (n = 31)	0.102 (4.57, 2)	Sham-IR	Non sig Kruskal-Wallis Test
		Sham-MI	
		IR-MI	
Day 1 (n = 31)	0.423 (1.72, 2)	Sham-IR	Non sig Kruskal-Wallis Test
		Sham-MI	
		IR-MI	
Day 2 (n = 31)	0.716 (0.67, 2)	Sham-IR	Non sig Kruskal-Wallis Test
		Sham-MI	
		IR-MI	
Day 7 (n = 28)	0.125 (4.16, 2)	Sham-IR	Non sig Kruskal-Wallis Test
		Sham-MI	
		IR-MI	
Day 14 (n = 27)	**0.035 (6.69, 2)**	Sham-IR	**0.037**
		Sham-MI	0.170
		IR-MI	1.00

Results to confirm that longitudinal strain fibrosis had higher standard deviation in the sham group when compared to radial strain measurement results

The standard deviation values of $PFM_{Longitudinal}$ were compared against corresponding values PFM_{Radial} using independent samples Mann-Whitney U test and the results were found to be significantly different with a *p-value* of 0.041.

Table S19: Comparison between standard deviation of PFM_{Radial} and $PFM_{Longitudinal}$ results in Sham Group

**Parameters**	Segments	N	Mean	Std. Deviation	Std. Error
PFM_{Radial}	1.00	7	5.2821	6.27886	2.37319
	2.00	8	3.5406	5.21039	1.84215
	3.00	7	4.0893	3.88970	1.47017
	4.00	7	3.6143	5.58897	2.11243
	5.00	7	1.1250	2.78066	1.05099
	6.00	8	3.3562	3.09299	1.09354
$PFM_{Longitudinal}$	1.00	7	9.8571	13.71435	5.18354
	2.00	8	1.8531	2.82572	0.99904
	3.00	7	18.5643	11.01646	4.16383
	4.00	7	14.4643	10.30738	3.89582
	5.00	7	19.8607	7.36502	2.78372
	6.00	8	13.9195	13.17339	4.65750
